# Towards an *in situ* non-lethal rapid test to accurately detect the presence of the nematode parasite, *Anguillicoloides crassus*, in European eel, *Anguilla anguilla*

**DOI:** 10.1017/S0031182021002146

**Published:** 2022-04

**Authors:** M. De Noia, R. Poole, J. Kaufmann, C. Waters, C. Adams, P. McGinnity, M. Llewellyn

**Affiliations:** 1 Institute of Biodiversity Animal Health and Comparative Medicine, University of Glasgow, G20 6NB, Glasgow, UK; 2 Marine Institute, Foras na Mara, F28 PF65, Newport, Ireland; 3 School of Biological, Earth and Environmental Sciences, University College Cork, T23 N73K, Cork, Ireland

**Keywords:** eDNA, fish parasite, genetic, non-lethal test

## Abstract

*Anguillicoloides crassus* is an invasive nematode parasite of the critically endangered European eel, *Anguilla anguilla*, and possibly one of the primary drivers of eel population collapse, impacting many features of eel physiology and life history. Early detection of the parasite is vital to limit the spread of *A. crassus*, to assess its potential impact on spawning biomass. However accurate diagnosis of infection could only be achieved via necropsy. To support eel fisheries management we developed a rapid, non-lethal, minimally invasive and *in situ* DNA-based method to infer the presence of the parasite in the swim bladder. Screening of 131 wild eels was undertaken between 2017 and 2019 in Ireland and UK to validate the procedure. DNA extractions and PCR were conducted using both a Qiagen Stool kit and *in situ* using Whatman qualitative filter paper No1 and a miniPCR DNA Discovery-System™. Primers were specifically designed to target the cytochrome oxidase mtDNA gene region and *in situ* extraction and amplification takes approximately 3 h for up to 16 individuals. Our *in-situ* diagnostic procedure demonstrated positive predictive values at 96% and negative predictive values at 87% by comparison to necropsy data. Our method could be a valuable tool in the hands of fisheries managers to enable infection control and help protect this iconic but critically endangered species.

## Graphical abstract

**Figure:**
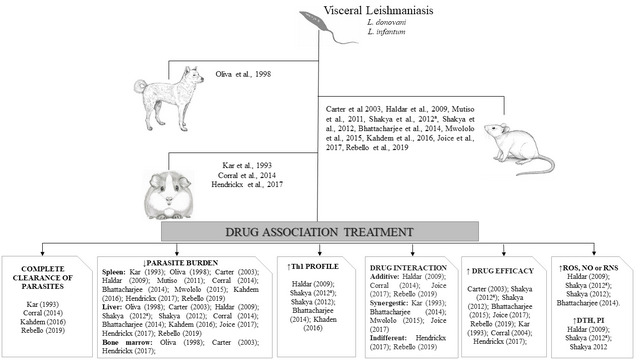

## Introduction

*Anguillicoloides crassus* (Kuwahara *et al.*, 1999) is a nematode parasite of the *Anguilla japonica* that also infects other *Anguilla* species, including the European eel *Anguilla anguilla* (Lefebvre *et al*., 2012)*. Anguillicoloides crassus* originates from East Asia, having been introduced into Europe in the early 1980s as a result of the trade in live Japanese eels, *A. japonica* (Temminck & Schlegel, 1847) (Laetsch *et al*., 2012; Weclawski *et al*., 2013). *Anguillicoloides crassus* is now well established in the Western Hemisphere and can be found in almost all European rivers and lakes, where it can tolerate salinities up to 12 ppt (Aguilar *et al*., 2005; Becerra-Jurado *et al*., 2014). While *A. crassus* was unlikely to have been the primary cause of the *A. anguilla* recruitment collapse since the 1980s, in conjunction with low recruitment, infections of the parasite may have contributed to declining adult stocks (Henderson *et al*., 2012) and to the quality of emigrating silver eels, thereby potentially impacting on effective spawner biomass and the ability of the stock to recover (Kirk, 2003).

*Anguillicoloides crassus* reproduces sexually in the swim bladder of the eels. The eggs hatch in the female worm inside the swim bladder and L2 larvae migrate to the intestinal tract *via* the pneumatic duct to be excreted with the feces (Didžiulis, 2013). As part of its life cycle, *A. crassus* is then trophically transmitted to various intermediate hosts including several zooplankton species (especially copepods of the orders Cyclopoidea and Calanoidea) as well as planktivorous fish such as the 3-spined sticklebacks, *Gasterosteus aculeatus* (Linnaeus, 1758) (Kuwahara and Itagaki, 1999). In the intermediate host, the parasite develops into the infectious phase L4 larvae, which, once ingested, parasitize the eel as the final host. The parasite migrates from the gut system perforating the connective tissue and muscles reaching the swim bladder (Heitlinger *et al*., 2009). The number of parasites found in the swim bladder can vary from <10 to >70 individuals per eel (Jousseaume *et al*., 2021). The presence of the parasite has been shown to detrimentally affect many features of eel physiology and life history (Newbold *et al*., 2015). Adult nematodes feed on blood supplied to the swim bladder wall and can result in increased eel mortality as a consequence of damage caused to the organ (Schneebauer *et al*., 2017). The swim bladder wall becomes thicker, opaque and less elastic due to the perforation caused by the parasite feeding habit with an impact on buoyancy control (Weclawski *et al*., 2013; Barry *et al*., 2014; Newbold *et al*., 2015). *Anguillicoloides crassus* infection is also thought to alter the physiological mechanisms involved in silvering – the process by which freshwater sub-adults adapt to life in the ocean. In this respect, infected eels have also been found to silver faster as a result of an over-production of cortisol, which seems to have a stimulatory effect on GTH2 synthesis (Muñoz *et al*., 2015; Di Biase *et al*., 2017). Moreover, cortisol is the key hormone produced during fasting, typical of the silvering phase stage (Fazio *et al*., 2012). During the silvering phase, a normal increase of erythropoiesis occurs, but the parasite, due to their blood feeding behaviour, increase erythropoiesis in infected eels prior their silvering (Churcher *et al*., 2015). The presence of the parasite may impact on the eel's migration speed in rivers (Newbold *et al*., 2015) and in the ocean as the energy demand increases (Pelster, 2015), due to the reduction of the swim bladder elasticity. The presence of the parasite appears not to affect the speed and migratory behaviour during the first phase of the migration in shallow water (Simon *et al*., 2018). However, where deep diving is required in the ocean, damage to the integrity of the swim bladder is believed to seriously impact on an infected eel's chances of survival (Lefebvre *et al*., 2012; Righton *et al*., 2016).

Currently accurate detection of the parasite can only be achieved *via* post-mortem dissection and thus requires the eel to be dissected. However, several non-lethal techniques are under development (Frisch *et al*., 2016). Anal redness can be used as an indicator for the presence or absence of the parasite, but this approach lacks both specificity and objectivity (Crean *et al*., 2003). A radio diagnostic method has been developed to detect inflammation caused by the nematode's feeding habits (Beregi *et al*., 1998). The method uses X-ray to scan the pneumatic duct and can detect swim bladder damage and parasite presence. The quality of the images has a large margin of error so the accuracy of detection can be low and swim bladder alterations can be caused by other factors (Beregi *et al*., 1998). Frisch *et al*. (2016) made improvements to the method developed by Beregi *et al*. (1998). Using compound radiography, they were able to detect small alterations to the thickness of the swim bladder wall and to inflations of the lumen. However, to perform a full body scan using this method, the animal is also to be euthanized. Recently, attempts have been made to develop a molecular test for *A. crassus* infection based in nuclear microsatellite markers (Jousseaume *et al*., 2021); however, reported sensitivity and specificity was below 71%, and the test, which involves fine-scale size discrimination of microsatellite locus sizes between target and off-target nematode species, is not easily transferred to field conditions. Finally, it is not clear from this molecular study whether feces could be sampled non-lethally (Jousseaume *et al*., 2021).

To support the assessment of eel stocks and ultimately fisheries management in the context of *A. crassus*, sensitive, specific and rapid non-lethal and *in situ* methods for pathogen detection are urgently required. Screening of translocated eel populations could, for example, limit the spread of the pathogen. Furthermore, non-lethal screening of silver eels alongside satellite tagging studies could reveal the impact of infection on the migratory and breeding success. Several non-lethal and/or molecular methods have been proposed to detect parasites in various fish species related to the food health safety chain and conservation management (Cavallero *et al*., 2017; Levsen *et al*., 2018). A non-lethal qPCR-based eDNA approach has also been optimized to detect the cestode *Schistocephalus solidus* in samples taken by needle from the intra-peritoneal cavity of a fish (Berger and Aubin-Horth, 2018).

In the current study, we developed an alternate, rapid, non-lethal and portable, *in situ* PCR-based approach to detect *A. crassus* in the European eel using parasite DNA traces in fecal material. We tested the specificity and sensitivity of 2 different DNA extraction methods, the former lab-based extraction protocol, the latter more suited to the field. Using necropsy data, we were also able to explore any link between host condition and parasite infection load.

## Materials and methods

### Sample collection

**Fig. 1.**
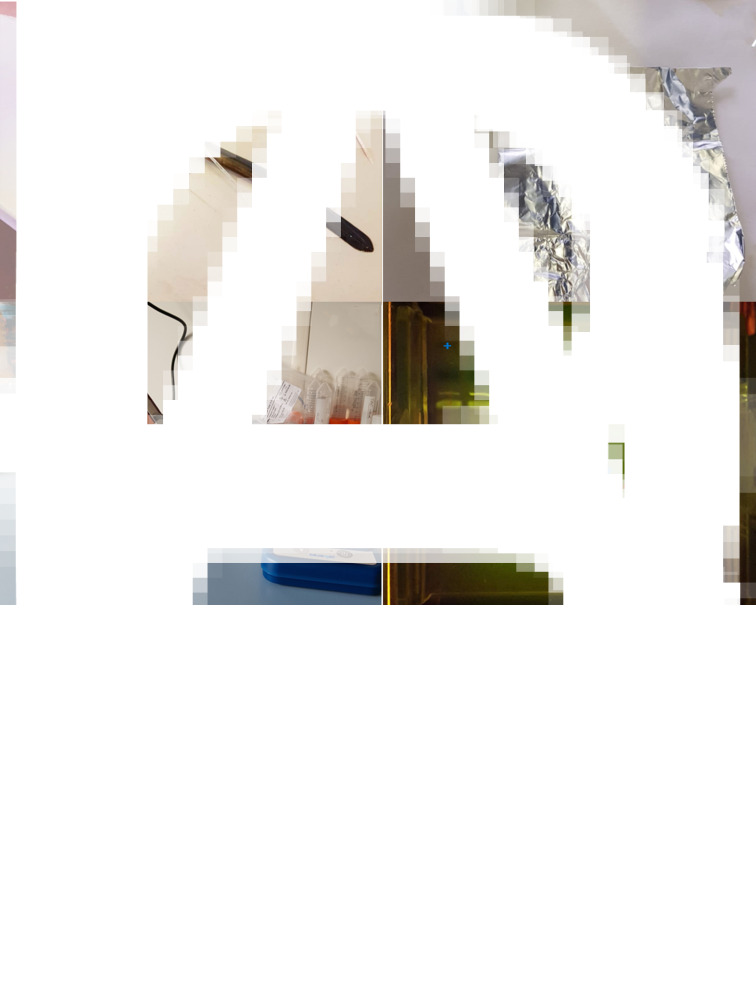
Experimental procedure for rapid, *in situ* and non-lethal molecular detection of *A. crassus* from the European eel. (A) Colonic irrigation with sterile saline solution (9%) on an anesthetized yellow eel. (B) Collection of a drop of fecal material on a piece of Whatman qualitative filter paper No. 1. (C) *In situ* DNA extraction and diagnostic PCR with miniPCR thermocycler. (D) *In situ* visualization on electrophoresis agarose gel 2% on amplified target CO1 gene. ‘+’ refers to positive amplification from fecal extracted DNA, ‘–’ negative amplification from fecal extracted DNA, ‘*’ positive control, ‘~’ negative control. The amplified fragment can be visualized around 187 bp. The band below represents resultant primer dimer.

The study was conducted at 2 different locations in the UK and Ireland. In the Burrishoole catchment, Ireland 53°55ʹ27.6ʺN 9°34ʹ27.0ʺW, yellow eels (feeding stage) were collected from Lough Feeagh (freshwater) and Furnace (tidal brackish water) using unbaited fyke nets deployed overnight in chains of 10 nets set at different lake depths in summer 2017, 2018 and 2019. Eels undergoing silvering were collected in autumn 2019 using permanent downstream river Wolf-type traps. The study was carried out under a Health Products Regulatory Authority (HPRA) license number AE19130-P096. In Lough Neagh, UK-Northern Ireland 54°36ʹ05.5ʺN 6°24ʹ55.5ʺW, yellow eels were collected with baited long lines fished overnight in the lake in summer 2018. Between capture and the procedures, eels were kept in holding tanks at the Marine Institute of the Burrishoole catchment. Under mild anaesthesia, colonic irrigation with 2 mL 0.09% sterile saline solution was performed to collect fecal material using a 5 mL syringe and a modified (needle removed) Terumo Surflo Winged Infusion Set (Fig. 1). Each colonic irrigation procedure lasted <30 s. The eels were then euthanized with an overdose of MS-222 (10 min in a 100 mg L^−1^ Tricaine methane sulfonate solution, FishVet Group (FVG) Ireland) followed by a cervical separation of the spinal cord. A total number of 131 eels were sampled and weight and length were recorded to the nearest 0.1 cm and gram (Supplementary Material 1). A drop of the collected wash material was placed on 1 cm^2^ of Whatman qualitative filter paper No. 1 and air dried for 5 min at room temperature. The air-dried paper was used to perform instant *in situ* DNA extraction or preserved at −80° C. The remaining wash was stored in 100% ethanol (1 wash: 9 ETOH) at −20°C. Subsequently, all eels were dissected, swim bladder inspected and the number of *A. crassus* present counted. *Anguillicoloides crassus* were collected and stored in 100% ethanol before being stored at −20° C. An eel was considered infected if at least one parasite, regardless of its lifecycle stage, was found in the swim bladder.

### DNA collection and extraction methods development

#### DNA extraction

A total of 131 eels were sampled and fecal material was collected from all. DNA was extracted from fecal wash of 104 eels using the Qiagen stool. The Whatman extraction protocol was used *in situ* for eels sampled in 2019 (*N* = 55). To enable direct comparison between the 2 protocols, DNA was extracted from 28 eels caught in 2019 (14 in Lough Furnace and 14 in Lough Feeagh) using both Qiagen and Whatman extraction methods for each eel. DNA concentrations for Qiagen extractions are included in Supplementary Table 3.

#### Laboratory genomic DNA extraction

DNA from 200 *μ*L of stored fecal material was centrifuged for 5 min at 12 000 rpm to concentrate the pellet. For each sample, 180 *μ*L of supernatant was removed and the remaining material was extracted with a slight modification to the suggested protocol of the QIAamp Stool Kit (Qiagen, Hilden, Germania). ATL tissue lysis buffer volume was increased to 350 *μ*L, proteinase K up to 20 *μ*L, AL lysis buffer up to 300 *μ*L and ethanol 100% up to 400 *μ*L.

#### *In situ* genomic DNA extraction

A small sample of the filter paper (where fecal material had been previously deposited) of 1 mm diameter was removed by a punch from the Whatman qualitative filter paper No. 1 and DNA was extracted using adjusted extraction protocol of DNA from Whatman™ FTA™ cards (Santos *et al*., 2006) (Fig. 1) (Supplementary Material 1).

### Primer design, PCR conditions and species specificity

A pair of specific customized primers were designed using all 467 cytochrome c oxidase subunit 1 (COI) gene sequences available for *A. crassus* (NCBI). All sequences were aligned to build a consensus sequence using BioEdit version 7.0.5.3 (Hall, 2017). The obtained consensus sequence was used to identify a conserved region within *A. crassus* suitable for primer design (Table 1). The designed primer pair was assayed for cross-reactivity *in silico* against common fish nematode parasites *Camallanus* sp. (NCBI Accession: EU598889), *Contracaecum* sp. (NCBI Accession: FJ866816) and *Capillaria* sp (NCBI Accession: AJ288168) (Pouder *et al*., 2009). The total length of the expected amplicon is 187 bp. The same PCR conditions and mastermix were used to test the efficiency and specificity of the primer using a miniPCR DNA Discovery System. The PCR Mastermix was made with 10 *μ*L of Q5® High-Fidelity DNA Polymerase, 1 *μ*L of FWD primer (10 nm), 1 *μ*L of RV primer (10 nm), 0.5 *μ*L MgCl_2_ (0.5 m), 6.5 *μ*L of RNA and DNA-free water and 1 *μ*L of extracted DNA. The total volume of the PCR reaction was 20 *μ*L per sample. The cycle used for the PCR started with 5 min at 95°C, followed by 35 cycles of 95°C for 30 s, 60°C for 30 s and 72°C for 30 s and a last step of 10 min at 72°C. In total, 5 *μ*L of PCR products was visualized on a 2% agarose gel using SYBR safe staining (Invitrogen). Each sample was amplified in technical triplicate alongside negative controls (ddH_2_0) and a positive control of either 20 ng *μ*L^−1^ DNA (Qiagen extraction) or *A. crassus* tissue crushed onto Whatman FTA card. Species specificity of the primer set was confirmed using a series of parasites collected in the same study system, various non-nematode parasites (*Anisakis* sp.) and other animal taxa, including the European eel, to assay cross-reactivity (*Lepeophtheirus salmonis*, *A. anguilla*, *Neoparamoeba perurans*, *Scomber scombrus*, *Diphyllobothrium* sp., *Schistocephalus* sp., *Dentitruncus truttae*). For each organism, 1 *μ*L of DNA was used. To identify amplicons as *A. crassus*, a subset of positive amplifications were Sanger-sequenced at MRC PPU DNA Sequencing and Services, Dundee, UK.

**Table 1.** Primer name, direction of amplification, primer size expressed in base pairs and specific designed sequence

Primer name	Direction	Primer size bp	Primer sequence
AcrasCO1_fwd	5′ -> 3′	26	CCATTCTGGTATAAGTGTTGATCTCG
AcrasCO1_rev	3′ -> 5′	30	ACAACCTCATATGTTCTARAGTAATAGAT

### Sensitivity, specificity, PPV and NPV

Several parameters were calculated to assay the validity of the test. Here *sensitivity* is defined as the ability of the test to correctly classify an individual as infected (i.e. a true positive). The ability of a test to correctly classify an individual as non-infected (i.e. a true negative) is called the test's *specificity*. The positive predictive value (PPV) is the percentage of eels with a positive test which are actually infected on dissection and the negative predictive value (NPV) is the percentage of eels with a negative test which do not have the parasite on dissection. PPV and NPV are directly related to the prevalence of the disease in the population (Stojanović *et al*., 2014) (Table 2).

**Table 2.** The criteria for specificity, sensitivity NPV and PPV as applied to a rapid test for *A. crassus*

	Dissection result			
Test result	Infected	Not infected		
Positive	True positive (TP)	False positive (FP)	Sensitivity = (TP)/(TP + FN)	PPV = (TP)/(TP + FP)
Negative	False negative (FN)	True negatives (TN)	Specificity = (TN)/(TN + FP)	NPV = (TN)/(TN + FN)

Animals that are infected and test positive are considered true positive (TP). Animals that are infected and test negative are described as false negative (FN). Animals that have no visible parasites, but test positive are false positive (FP), and those that have no visible parasites but test negative are true negative (TN).

NPV, negative predictive value; PPV, positive predictive value.

### Biological validation of eel infection status

#### Eggs count in fecal wash

Nematode larvated and unlarvated eggs and L2 larvae were counted with a modified McMaster Salt Flotation Technique. In total, 200 *μ*g of fecal material was diluted in 1.5 mL of distilled water. After mechanical homogenization, the suspension was poured through a 250-micron aperture sieve and the filtrate collected. After thorough mixing, the solution was transferred to a centrifuge tube and spun for 5 min at 2500 rpm. The supernatant was discarded and the remaining fecal pellet covered and homogenized with 300 *μ*L of saturated sodium chloride solution, mixed by inverting slowly 6 times. Then, using a Pasteur pipette, the mixture was transferred to a McMaster slide. Each chamber holds 0.15 mL beneath the gridded area. The preparation is then examined using the 25× objective of a stereoscopic microscope, and the number of eggs present in the grids of both chambers was counted to give an estimate of the numbers of eggs per gram of fecal material.

#### *Anguillicoloides crassus* count in swim bladder necropsy

All dissected eels were checked for *A. crassus*, and where present, they were counted. The swim bladder was extracted whole from the animal and stored at 4°C until the procedure was completed for all the specimens. The swim bladder was then opened and nematodes were counted and classified to adults and larval stages. A Mann–Whitney test was performed in R studio between years of infection to test if there was significant change in the parasitic load.

## Results

### Rapid test *in situ*

The *in situ* non-lethal test was performed on 55 eels collected in 2019. Individuals were anally catheterized to enable colonic irrigation with a soft silicon tube without causing internal lesions. The amount of saline solution used (between 0.5 and 2 mL) varied in approximate proportion to the size of the tested animals. The procedure was deployed to minimize the invasiveness of the collection of the fecal material. *In situ* DNA extraction took 20 min for 16 samples, PCR reaction was undertaken over a period of 2 h and electrophoresis with gel visualization took a further 17 min. Thus, the test can be performed for 16 individuals in approximately 3 h (Fig. 1).

### Comparison of different DNA extraction methods

**Fig. 2.**
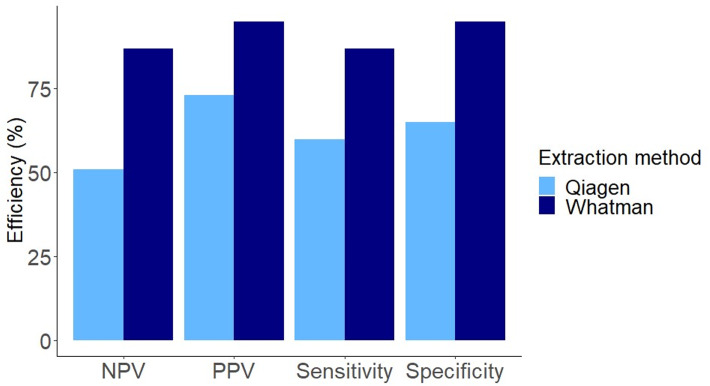
Relative *A. crassus* parasite detection efficiency for the 2 DNA extraction methods. The Whatman DNA extraction method (dark blue bar) performs better in all the categories with an average improvement of 41% over the Qiagen method (pale blue bar). A Welch 2-sample *t*-test indicates both sensitivity (*P* < 0.006) and specificity (*P* < 0.003) were significantly improved by using the Whatman protocol. NPV, negative predictive value; PPV, positive predictive value.

The DNA Whatman paper extraction method provides a rapid and more reliable assessment of infection compared to the method based on the Qiagen Stool and Blood kit. Both specificity (*P* < 0.006) and sensitivity (*P* < 0.003) were shown to be significantly higher using the Whatman protocol (Fig. 2, Table 3). The resulting improvement of using the Whatman test in specificity was 46%, in sensitivity 45%, in PPV 30% and in NPV 41% (Fig. 2). Additionally, the time for DNA extraction from 1 sample using the Whatman paper as compared to a Qiagen extraction was reduced from c.80 to c.20 min.

**Table 3.** Relative *A. crassus* detection for the 2 different extraction methods across the 3 sampling seasons

Extraction method	Year	No. samples	Observed infected	Test positive	Test negative	False positive	False negative
Qiagen	2017	12	8	3	2	3	4
Qiagen	2018	23	12	4	8	2	9
Qiagen	2018n	41	28	20	10	4	7
Qiagen	2019	28	18	16	4	6	2
Whatman paper	2019	28	18	19	9	2	1
Whatman paper	2019s	27	23	22	4	0	1

For 2019 the same samples were tested with both methods. 2018n considers samples collected in Lough Neagh and 2019s silver eels collected in the Burrishoole catchment.

### Parasite count in swim bladder and eggs count in fecal material

**Fig. 3.**
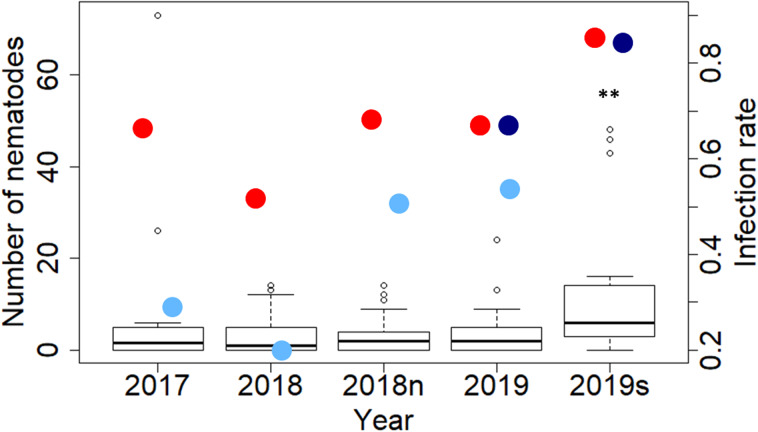
Number of *A. crassus* counted in dissected animals and infection rate in different years of sampling. Infection prevalence represents the number of animal infected compared to the total number of animals. Dark line in each box stands for the mean number of nematode per cohort of sampling. Red dots show the actual infection rate based on average parasite load in dissected animals, each empty dot stands for a single dissected eel. Light blue dots indicate the infection rate derived from the extraction using Qiagen Blood and Stool kit. Dark blue dots indicate the infection rate observed with Whatman. A Mann–Whitney test shows that silver eels in 2019 were significantly more infected than other eels (*P* < 0.05). ‘2018n’ refers to samples collected in Lough Neagh and ‘2019s’ to silver eels collected in the Burrishoole system.

Fecal material from all 131 samples was tested to detect the presence of eggs and/or L2 larvae using the McMaster floatation protocol. All 131 collected swim bladders were then screened under the microscope and number of nematode eggs reported (Supplementary Material 1). No eggs or larvae were detected in any samples. Number of nematodes in the swim bladder showed an increasing trend in infection across the 3 sampling years and a significant increase in the silver eels collected in 2019 (Fig. 3).

## Discussion

Our study represents the first attempt to develop a sensitive, non-lethal and, importantly, *in situ* method to establish *A. crassus* infection in *A. anguilla via* the detection of parasite DNA in fecal material. High values for NPV (87%) and PPV (95%) suggest the test may have a useful role in both veterinary and fisheries management contexts. We found inter-annual differences in the prevalence of infected eels in the 3 years we sampled in the Burrishoole catchment with the total number of infected animals significantly higher in 2019.

Sensitivity is a key consideration for any molecular test. Mitochondrial genes are the major target genes in PCR-based detection systems because they are highly conserved and present in multiple copies (Paoletti *et al*., 2018). High target copy number may explain the high sensitivity of the test we deployed. The mitochondrial gene COI has been widely used to detect the presence of nematode parasites in commercially important fish species (Santos *et al*., 2006; Herrero *et al*., 2011; Godínez-González *et al*., 2017; Paoletti *et al*., 2018). The use of microsatellites is also a well-established method for parasite detection (Vieira *et al*., 2016), although these nuclear markers can suffer from lower sensitivity than their mitochondrial counterparts, which may contribute to their poorer performance in detecting *A. crassus* in eels (Jousseaume *et al*., 2021).

Some improvement in detecting sensitivity and specificity was achieved here by adopting a more ‘crude’ nucleic acid extraction approach using a Whatman paper. Nucleic acid extraction is increasingly recognized as a major rate limiting step in molecular diagnostics; however, paper-based options offer several advantages in terms of speed and cost (Zou *et al*., 2017), as seems to be the case in our study. Nonetheless, our final protocol did show both false positives and false negatives, albeit at a low rate. False negatives likely relate to issues with template purification and PCR amplification, or potentially the reduced biomass of younger worms (Barry *et al*., 2014). Similarly juvenile, un-mated worms are likely to shed less genetic material in the form of larvae. False positives could indicate the presence of early infections, not yet detectable *via* necropsy – and further investigation of such cases is warranted. It is not clear whether the DNA we are detecting originates from embryonated eggs, cellular material or free DNA shed from the worms. Our inability to microscopically detect the presence of *A. crassus* larvae or eggs in fecal material suggests that the DNA or cellular fragments from worms are the likely source. However, our use of ethanol as a fixative for storing samples could have played a role in our low success in detecting eggs or larvae (Crawley *et al*., 2016).

The test we present relies on a simple PCR, not qPCR. Nonetheless, validation against ‘real’ infection levels assayed *via* necropsy reveals excellent specificity and sensitivity. Point-of-care qPCR for viral pathogens can now deliver a result in <20 min (Melchers *et al*., 2017). Similarly, several mobile qPCR instruments have been brought to the market and have been successfully deployed to deliver veterinary diagnoses in remote locations on actionable timescales for cattle (Hole and Nfon, 2019). However, the cost of such approaches may be prohibitive in respect to their application to the detection of pathogens fish. Our experimental set-up, which fits in a carry-on suitcase and can be performed in the field powered with a portable battery, shows that standard PCR, using low-cost reagents and equipment, may be just as portable, and informative epidemiologically, as ‘higher-end’ devices, although benchmarking against a portable qPCR device could be a focus of future study.

Stocking, as part of eel population enhancement, is likely a major contributor to *A. crassus* dispersal, as are translocations associated with the trade in live eels (Laetsch *et al*., 2012; Weclawski *et al*., 2013). Screening of such individuals and early detection with a non-lethal method could be a powerful tool to avoid the spread of the parasite. However, there remains a need to clarify whether our approach has enough sensitivity to detect infection in glass eels and elvers. *Anguillicoloides crassus* is known to infect the elvers or European eels (Haenen *et al*., 1989) and natural infection has been detected in late-stage glass eels as well as elvers of the American eel *Anguilla rostrata* (Hein *et al*., 2016). Both juvenile stages are a major component of stocking biomass. Advances in sample pooling designs and detection algorithms during the recent coronavirus epidemic can achieve individual-level identification using a 7-fold lower number of tests than the number of individuals (Shental *et al*., 2020). Such algorithms could also be adapted to screen large cohorts of eels, but regulation and legislation may be required before industry agrees to bear the associated cost.

The increase in parasite load we noted from 2017 to 2019 follows a trend that is also found all over Europe, where the parasite is established and is fast colonizing all the freshwater basins (Aguilar *et al*., 2005; Schabuss *et al*., 2005; Wielgoss *et al*., 2008; Selim and El-ashram, 2012). *Anguillicoloides crassus* has a very recent history in the Burrishoole catchment. First detection occurred in 2010 in a yellow eel in brackish water and in 2016 for the first time in a silver eel from freshwater (R. Poole, personal communication). In contrast to the rising burdens across much of Europe, in some lakes where the parasite had been detected since first discovery, there is stabilization and even a slight decline in nematode abundance and intensities (Wielgoss *et al*., 2008). There is a possibility of an increased resistance towards the parasites in the long term (Schabuss *et al*., 2005). Although some evidence of increasing tolerance of *A. anguilla* to parasite infection, the overall impact of the parasite on the eel's mortality has been severe and is likely a contributor to the European population's steep decline impeding a recovery (Molnar *et al*., 1993; Kirk, 2003; Schabuss *et al*., 2005). Treatment of infected eels with anti-helminthic has not been trialled, and a single vaccination study aimed at reducing the development of adult from irradiated L3 larvae was unsuccessful and revealed the antibody response is not a key element in the resistance of *A. anguilla* against *A. crassus* (Knopf and Lucius, 2008). Infection control *via* physically blocking transmission, which requires extensive diagnostic testing, therefore represents the only feasible route to reducing population-wide parasite burden.

In this study, we developed potentially useful tool that can be deployed for specific parasite screening for the European eel. Cost per sample is low and the time to run a test comprising 16 samples is under 3 h. Our test offers managers the opportunity to engage in infection control by assessing the disease status of adult eels before allowing transfers between river systems, although further work is required to establish whether it can survey juveniles. Nonetheless, the rapid test represents an important contribution to the conservation and management of this critically endangered species.

## Data Availability

All data generated or analysed during this study are included in this published article. The dataset supporting the conclusions of this article is available in the Supplementary material section. Supplementary Table 1 reports all the data collected, Supplementary Table 2 the DNA extraction protocol and Supplementary Table 3 the DNA concentration of the fecal material extracted with the Qiagen method.

## Supplementary material

For supplementary material accompanying this paper visit https://doi.org/10.1017/S0031182021002146. click here to view supplementary material
