# Supplementary material for: Towards an in situ non-lethal rapid test to accurately detect the presence of the nematode parasite, Anguillicoloides crassus, in European eel, Anguilla anguilla
Source: Parasitology. 2022 Jan 19;149(5):605–11. doi: 10.1017/S0031182021002146 (PMC10090626; doi:10.1017/S0031182021002146)
Supplement: Supplementary file 1 [file S0031182021002146sup.zip › S0031182021002146sup003.docx]

Supplementary 3. DNA concentration measured with Thermo Scientific™ NanoDrop 2000 for each samples extracted with Qiagen method.

| Eel | Name | Year | Concentration [ng/µl] | 260/280 | 260/230 |
| --- | --- | --- | --- | --- | --- |
| 1 | Ang17_0050 | 2017 | 2.7 | 1.06 | 1.09 |
| 2 | Ang17_0051 | 2017 | 4.1 | 4.4 | 0.68 |
| 3 | Ang17_0052 | 2017 | 6.2 | 2.01 | 0.41 |
| 4 | Ang17_0053 | 2017 | 6.3 | 2.04 | 0.54 |
| 5 | Ang17_0059 | 2017 | 5.1 | 10.6 | 0.56 |
| 6 | Ang17_0062 | 2017 | 2 | 1.78 | 0.31 |
| 7 | Ang17_0079 | 2017 | 23.2 | 1.81 | 2.04 |
| 8 | Ang17_0080 | 2017 | 31 | 5.76 | 0.78 |
| 9 | Ang17_0081 | 2017 | 5.3 | 1.51 | 0.45 |
| 10 | Ang17_0082 | 2017 | 4.1 | 3.05 | 0.41 |
| 11 | Ang17_0083 | 2017 | 6 | 3.15 | 0.68 |
| 12 | Ang17_0085 | 2017 | 5.5 | 0.71 | 0.91 |
| 13 | ANG18_1000 | 2018 | 1.5 | 3.82 | 1.01 |
| 14 | ANG18_1001 | 2018 | 2.4 | 0.69 | 0.5 |
| 15 | ANG18_1002 | 2018 | 0.2 | 0.78 | 0.81 |
| 16 | ANG18_1003 | 2018 | 1.8 | 1.02 | 0.74 |
| 17 | ANG18_1004 | 2018 | 1.4 | 0.22 | 1.05 |
| 18 | ANG18_1005 | 2018 | 0.4 | 1.24 | 2.7 |
| 19 | ANG18_1006 | 2018 | 1.4 | 0.78 | 0.32 |
| 20 | ANG18_1007 | 2018 | 2.6 | 1.83 | 1.75 |
| 21 | ANG18_1008 | 2018 | 4.3 | 0.53 | 0.9 |
| 22 | ANG18_1009 | 2018 | 3 | 1.9 | 1.57 |
| 23 | ANG18_1010 | 2018 | 2.5 | 2.73 | 7.32 |
| 24 | ANG18_1011 | 2018 | 1.1 | 0.43 | 0.89 |
| 25 | ANG18_1012 | 2018 | 3.2 | 1.92 | 1.43 |
| 26 | ANG18_1013 | 2018 | 3.3 | 1.48 | 3.87 |
| 27 | ANG18_1018 | 2018 | 12.4 | 1.9 | 1.84 |
| 28 | ANG18_1019 | 2018 | 0.6 | 0.33 | 0.76 |
| 29 | ANG18_1020 | 2018 | 0.8 | 0.69 | 0.94 |
| 30 | ANG18_1023 | 2018 | 5.3 | 1.24 | 1.89 |
| 31 | ANG18_1024 | 2018 | 20.3 | 1.65 | 2.65 |
| 32 | ANG18_1025 | 2018 | 7.2 | 1.83 | 3.57 |
| 33 | ANG18_1029 | 2018 | 0.8 | 1.79 | 2.95 |
| 34 | ANG18_1030 | 2018 | 0.8 | 2.75 | 1.46 |
| 35 | ANG18_1034 | 2018 | 1 | 0.46 | 0.71 |
| 36 | ANG19_40 | 2019 | 2.6 | 2.36 | 2.45 |
| 37 | ANG19_41 | 2019 | 0.8 | 0.92 | 0.41 |
| 38 | ANG19_42 | 2019 | 0.3 | 0.9 | 0.81 |
| 39 | ANG19_43 | 2019 | 0.5 | 0.89 | 0.88 |
| 40 | ANG19_44 | 2019 | 2.9 | 0.47 | 1.62 |
| 41 | ANG19_45 | 2019 | 3.8 | 1.78 | 1.43 |
| 42 | ANG19_46 | 2019 | 0.2 | 2.3 | 2.54 |
| 43 | ANG19_47 | 2019 | 0.9 | 2.71 | 4.34 |
| 44 | ANG19_48 | 2019 | 17.3 | 7.31 | 1.98 |
| 45 | ANG19_49 | 2019 | 2.7 | 0.44 | 6.41 |
| 46 | ANG19_50 | 2019 | 14.1 | 1.36 | 1.82 |
| 47 | ANG19_51 | 2019 | 0.5 | 0.77 | 0.38 |
| 48 | ANG19_63 | 2019 | 2.4 | 0.91 | 1.43 |
| 49 | ANG19_64 | 2019 | 0.4 | 1.76 | 1.1 |
| 50 | ANG19_65 | 2019 | 15.2 | 3.54 | 4.56 |
| 51 | ANG19_66 | 2019 | 7.4 | 2.2 | 1.9 |
| 52 | ANG19_67 | 2019 | 1.3 | 1.21 | 1.23 |
| 53 | ANG19_68 | 2019 | 1.2 | 1.91 | 1.21 |
| 54 | ANG19_69 | 2019 | 1.3 | 1.08 | 1.01 |
| 55 | ANG19_70 | 2019 | 0.7 | 0.53 | 0.71 |
| 56 | ANG19_71 | 2019 | 1.2 | 1.31 | 1.45 |
| 57 | ANG19_72 | 2019 | 1.6 | 1.2 | 1.32 |
| 58 | ANG19_73 | 2019 | 3.3 | 2.21 | 4.4 |
| 59 | ANG19_74 | 2019 | 3.7 | 0.45 | 0.43 |
| 60 | NE2018_1 | 2018N | 84 | 1.96 | 1.73 |
| 61 | NE2018_2 | 2018N | 153 | 2.03 | 1.88 |
| 62 | NE2018_3 | 2018N | 19 | 0.86 | 0.39 |
| 63 | NE2018_4 | 2018N | 74 | 1.91 | 1.92 |
| 64 | NE2018_5 | 2018N | 10.2 | 1.57 | 8.32 |
| 65 | NE2018_6 | 2018N | 73.5 | 1.77 | 2.52 |
| 66 | NE2018_7 | 2018N | 5.6 | 1.37 | 2.27 |
| 67 | NE2018_8 | 2018N | 5.3 | 1.83 | 0.53 |
| 68 | NE2018_9 | 2018N | 3.3 | 1.53 | 3.62 |
| 69 | NE2018_10 | 2018N | 58.7 | 1.57 | 0.35 |
| 70 | NE2018_11 | 2018N | 10.3 | 1.96 | 1.75 |
| 71 | NE2018_12 | 2018N | 14.8 | 1.75 | 4.28 |
| 72 | NE2018_13 | 2018N | 18.6 | 1.87 | 3.28 |
| 73 | NE2018_14 | 2018N | 47.2 | 1.97 | 1.86 |
| 74 | NE2018_15 | 2018N | 22.9 | 1.65 | 3.62 |
| 75 | NE2018_16 | 2018N | 68.2 | 1.82 | 1.87 |
| 76 | NE2018_17 | 2018N | 40.1 | 2.01 | 1.45 |
| 77 | NE2018_18 | 2018N | 12.1 | 2.5 | 1.74 |
| 78 | NE2018_19 | 2018N | 43.5 | 2.41 | 3.62 |
| 79 | NE2018_20 | 2018N | 65.4 | 1.03 | 2.7 |
| 80 | NE2018_21 | 2018N | 0.9 | 0.76 | 1.73 |
| 81 | NE2018_22 | 2018N | 1.4 | 0.91 | 0.77 |
| 82 | NE2018_23 | 2018N | 3.2 | 1.43 | 1.23 |
| 83 | NE2018_24 | 2018N | 19.7 | 1.54 | 1.9 |
| 84 | NE2018_25 | 2018N | 67.4 | 1.62 | 2.21 |
| 85 | NE2018_26 | 2018N | 43.7 | 1.73 | 2.4 |
| 86 | NE2018_27 | 2018N | 92.1 | 1.98 | 4.21 |
| 87 | NE2018_28 | 2018N | 8.2 | 2.9 | 2.5 |
| 88 | NE2018_29 | 2018N | 41.1 | 0.43 | 0.66 |
| 89 | NE2018_30 | 2018N | 16.4 | 0.96 | 0.9 |
| 90 | NE2018_31 | 2018N | 74.2 | 2.98 | 3.81 |
| 91 | NE2018_32 | 2018N | 32.4 | 2.75 | 0.57 |
| 92 | NE2018_33 | 2018N | 9.3 | 1.93 | 2.04 |
| 93 | NE2018_34 | 2018N | 13.3 | 1.95 | 4.21 |
| 94 | NE2018_35 | 2018N | 0.3 | 1.43 | 1.76 |
| 95 | NE2018_36 | 2018N | 75.4 | 1.89 | 1.5 |
| 96 | NE2018_37 | 2018N | 6.4 | 2.7 | 1.33 |
| 97 | NE2018_38 | 2018N | 92.3 | 2.35 | 4.43 |
| 98 | NE2018_39 | 2018N | 65.5 | 2.91 | 0.31 |
| 99 | NE2018_40 | 2018N | 21.4 | 2.08 | 2.57 |
| 100 | NE2018_41 | 2018N | 5.4 | 0.54 | 0.65 |
| 101 |  |  |  |  |  |
| 102 |  |  |  |  |  |
| 103 |  |  |  |  |  |
| 104 |  |  |  |  |  |
| 105 |  |  |  |  |  |
| 106 |  |  |  |  |  |
| 107 |  |  |  |  |  |
| 108 |  |  |  |  |  |
| 109 |  |  |  |  |  |
| 110 |  |  |  |  |  |
| 111 |  |  |  |  |  |
| 112 |  |  |  |  |  |
| 113 |  |  |  |  |  |
| 114 |  |  |  |  |  |
| 115 |  |  |  |  |  |
| 116 |  |  |  |  |  |
| 117 |  |  |  |  |  |
| 118 |  |  |  |  |  |
| 119 |  |  |  |  |  |
| 120 |  |  |  |  |  |
| 121 |  |  |  |  |  |
| 122 |  |  |  |  |  |
| 123 |  |  |  |  |  |
| 124 |  |  |  |  |  |
| 125 |  |  |  |  |  |
| 126 |  |  |  |  |  |
| 127 |  |  |  |  |  |
| 128 |  |  |  |  |  |
| 129 |  |  |  |  |  |
| 130 |  |  |  |  |  |
| 131 |  |  |  |  |  |
